# Supplementary material for: Impact of Poloxamer on Crystal Nucleation and Growth of Amorphous Clotrimazole
Source: Pharmaceutics. 2023 Aug 21;15(8):2164. doi: 10.3390/pharmaceutics15082164 (PMC10460058; doi:10.3390/pharmaceutics15082164)
Supplement: Supplementary file 1 [file pharmaceutics-15-02164-s001.zip › pharmaceutics-2536707-supplementary.pdf]

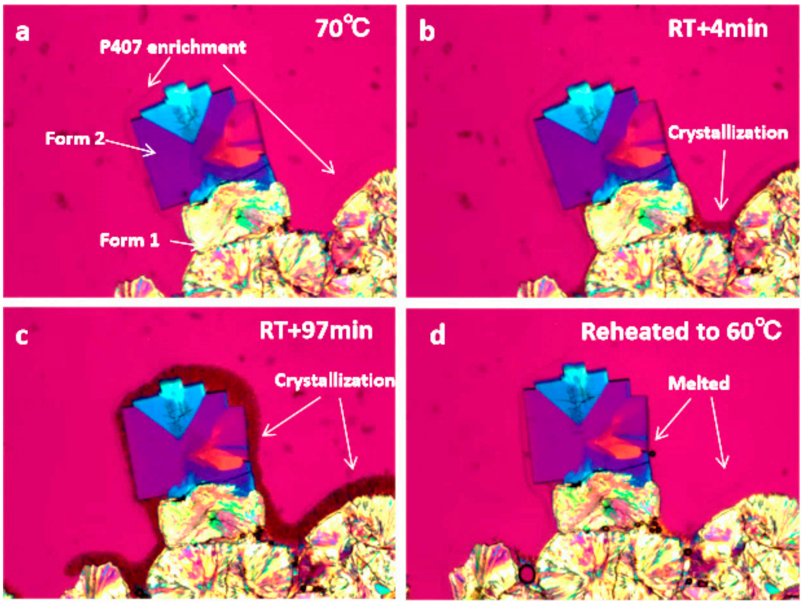

Figure S1. Photomicrographs of CMZ crystals grown in the presence of 10% w/w PEO at 70 ℃ (a); cooled to 25 ℃ and held for 4 min (b); cooled to 25 ℃ and held for 97 min (c); and re-heated to 60 ℃ (d).

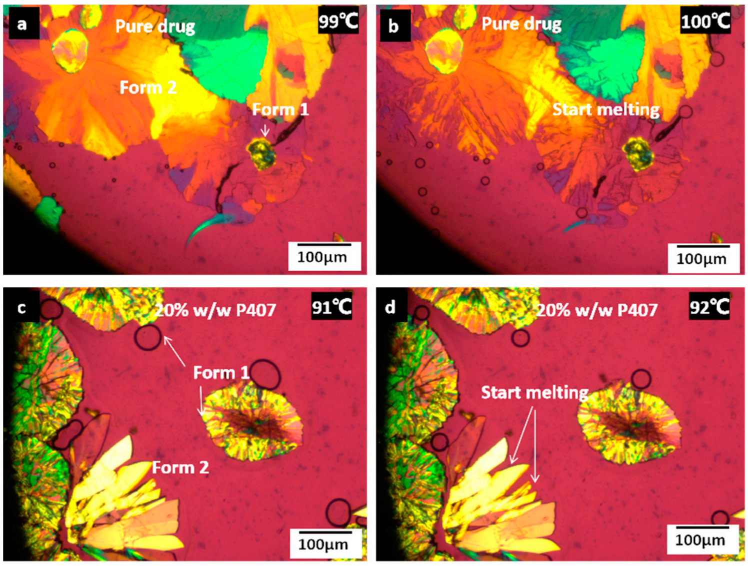

Figure S2. The polarized light microscopic illustration for Form 2 melting upon heating in pure drug (a,b); in the presence of 20% w/w PEO at 70 ℃ (c,d).

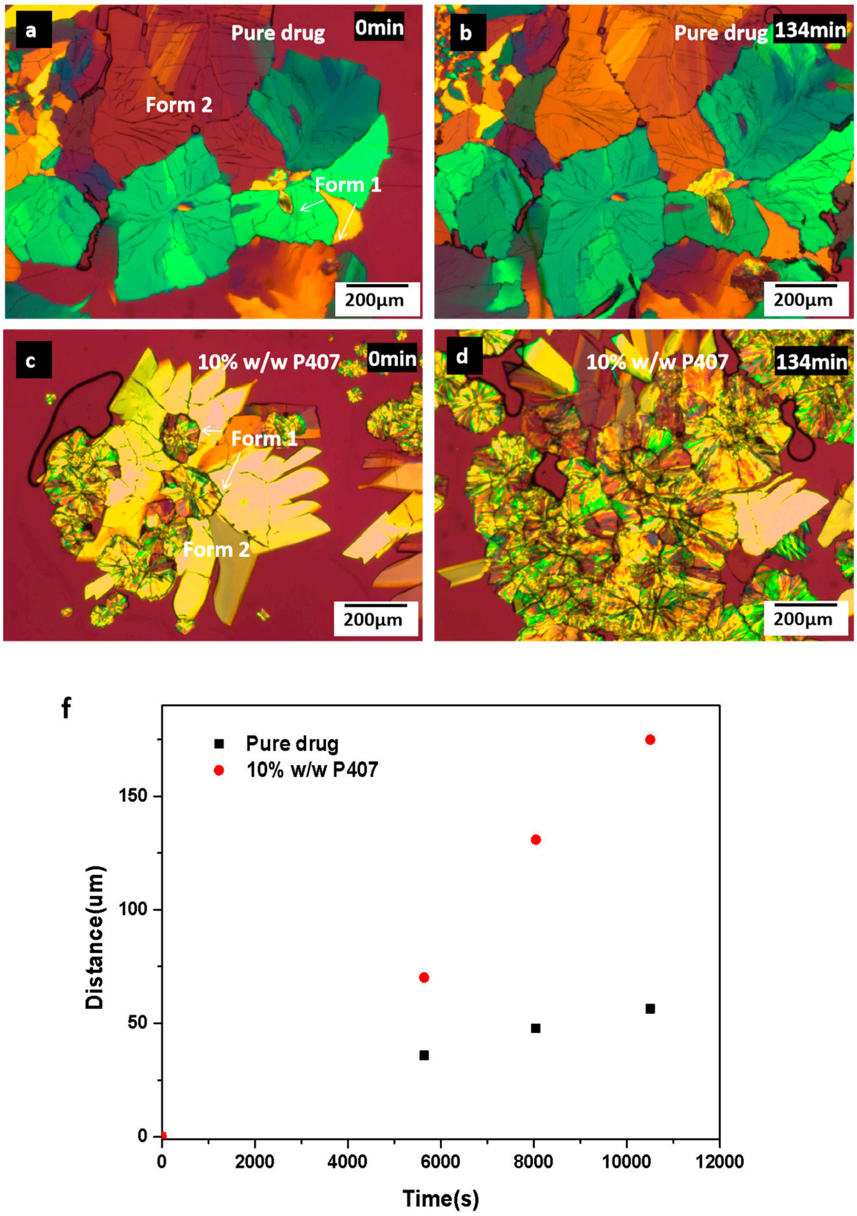

Figure S3. Photomicrographs of crystal transformation in pure drug (a,b) and in the presence of 10% w/w PEO at 70 ℃ (c,d) ; Typical data of transformation distance vs. time (f).The data are shifted vertically to coincide at the origin for better comparison.
